# Terpenes extracted from marine sponges with antioxidant activity: a systematic review

**DOI:** 10.1007/s13659-023-00387-y

**Published:** 2023-08-09

**Authors:** Cintia Cristina Santi Martignago, Beatriz Soares-Silva, Julia Risso Parisi, Lais Caroline Souza e Silva, Renata Neves Granito, Alessandra Mussi Ribeiro, Ana Cláudia Muniz Renno, Lorena Ramos Freitas de Sousa, Anna Caroline Campos Aguiar

**Affiliations:** 1 Departamento de Biociências, Universidade Federal de São Paulo (UNIFESP), Rua Silva Jardim 136, Edifício Central, Santos, SP 11015-020 Brazil; 2 Institute of Chemistry, Federal University of Catalão (UFCAT), Av. Dr. Lamartine Pinto de Avelar, 1120 Vila Chaud, Catalão, GO 75704-020 Brazil

**Keywords:** Antioxidant, Marine drugs, Sponge, Free radical

## Abstract

Marine biodiversity has emerged as a very promising resource of bioactive compounds and secondary metabolites from different sea organisms. The sponge’s secondary metabolites demonstrated various bioactivities and potential pharmacological properties. This systematic review of the literature focuses on the advances achieved in the antioxidant potential of marine sponges in vitro. The review was performed in accordance with PRISMA guidelines. The main inclusion criterion for analysis was articles with identification of compounds from terpene classes that demonstrate antioxidant activity in vitro. Searching in three different databases, two hundred articles were selected. After screening abstracts, titles and evaluating for eligibility of manuscripts 14 articles were included. The most performed analyzes to detect antioxidant activity were scavenging activity 2,2-diphenyl-1-picrylhydrazyl (DPPH) and measurement of intracellular reactive oxygen species (ROS). It was possible to identify 17 compounds of the terpene class with pronounced antioxidant activity in vitro. Scientific evidence of the studies included in this review was accessed by the GRADE analysis. Terpenes play an important ecological role, moreover these molecules have a pharmaceutical and industrial application.

**Graphical Abstract:**

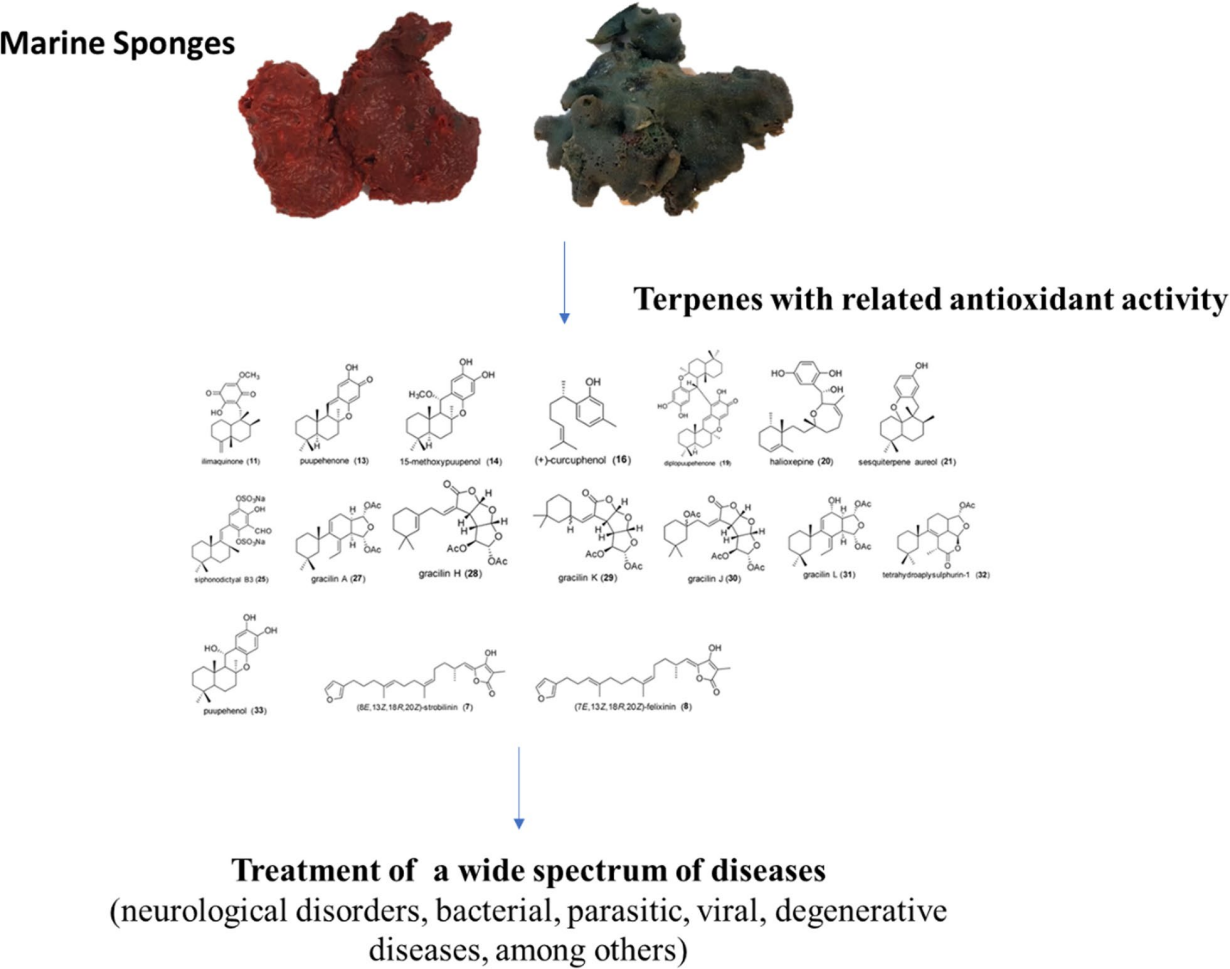

**Supplementary Information:**

The online version contains supplementary material available at 10.1007/s13659-023-00387-y.

## Introduction

Most living organisms on earth thrives in marine environments, providing a plentiful resource of potential novel products for society. Different species in a range of phyla have been studied under a biotechnological perspective, including marine sponges, which are known to be extremely rich in natural bioactive compounds [1].

Marine sponges (phylum Porifera) are sessile, colonial and aquatic organisms with an intense filter-feeding habit. Furthermore, in order to survive in marine environment, sponges produce many secondary metabolites. Moreover, these organisms typically host a huge variety of microbial communities which constitute around 35% of their total body mass [2]. These microbes, in turn, take on important roles within their host, supporting health and adaptability features, contributing to the production of second metabolites that can protect sponges against predators or pathogens [3, 4].

The secondary metabolites from sponges showed potential pharmacological properties, some of them currently on the market and in clinical trials, being used as drugs with anti-cancer, immunomodulatory, analgesic or anti-inflammatory effects [5]. In addition, isolated bioactive compounds from sponges with anticancer effects showed to prevent tumor formation and growth, and inducing cell death by apoptosis [6, 7]. For example, the compound eribulin mesylate (a macrolide polyketide), extracted from marine sponges, was approved for the treatment of metastatic breast cancer [8]. Moreover, metabolites from sponges with anti-malarial and anti-fungal effects, for example bromopyrrole alkaloids have showed promising outcomes such as anticancer drug [9–11]. Bioactive substances with anti-inflammatory and antioxidant properties are among the most important compounds of marine sponges [12].

Antioxidants are known to play an important role in preventing the development and treatment of pathologies such as cancer and cardiovascular diseases [13]. Terpenes are a class of organic compounds found in a wide variety of organisms, including plants, animals, and microorganisms. They are formed by the combination of basic units of isoprene, which are five-carbon structures. Terpenes play various important biological functions, such as being essential components of essential oils, plant pigments (such as carotenoids), steroid hormones, and precursors to bioactive molecules. Metabolites consisting of benzenoid (phenol or quinoid) and terpenoids are some of the most active marine metabolites, with antioxidant potential and their effects have been demonstrated in some in vitro studies [14–19]. For example, Rivera et al. investigated the antioxidant effects of hexane, dichloromethane, and ethyl acetate extracts of 5 different marine sponge species (*Aaptos suberitoides*, *Dactylospongia elegans*, *Stylissa massa*, *Haliclona* sp. and an unidentified species) and coded as KL-05 [20]. The authors demonstrated the antioxidant potential of the compounds, which were classified as alkaloids, saponins, tannins, and flavonoids [20]. Moreover, Araujo et al. [21] demonstrated the antioxidant activity in vitro of a collagenous compound extracted from the marine sponges *Chondrilla caribensis*.

Furthermore, while there is evidence supporting the manifestation of pharmacologically valuable components, particularly antioxidant compounds obtained from marine sponges, there is still a limited understanding of their biological effects. In this context, the purpose of the present study is to perform a systematic review of the literature to survey works investigating the antioxidant potential of compounds from marine sponges in vitro studies.

## Results

Two hundred articles were retrieved from the following database: 25 from Embase; 43 from PubMed and 132 from Scopus. Fifty-four duplicated articles were excluded, and 146 articles were included. Thirty-eight articles were excluded by reading the title and 78 by reading the abstract. Then, 30 articles were read in full and from those, 16 were excluded (6 papers have not evaluated isolated compounds from terpenes class, 4 did not evaluate antioxidant activity, 2 studies did not include terpenes, 2 investigated terpenes from another marine species, 1 was performed for validation of computer software and other 1 was not available for download). Thus, 14 articles were included in the present review (flow diagram in Additional file 1: Fig. S1). Scientific evidence was synthesized using the GRADE approach. The analysis of the data for each domain of the GRADE approach, the studies and the reasons for downgrading the quality of evidence is presented in Additional file 1: Table S1. The quality of evidence was moderate for studies that used 2,2-diphenyl-1-picrylhydrazyl (DPPH) and reactive oxygen species (ROS) analyses.

The most frequent collection site was the Fiji sea [15, 22]. Other sites included Korea [16]; Australia [15]; Indonesia [17]; Mexico [23]; Hawaii [24]; Taiwan [25]; Italy [26] and United States of America [27]. However, five studies did not report the location of the sponge collection [14, 18, 25, 28]. Three articles also referred to the geographic coordinate of the site collected [15, 22, 24].

The included studies evaluated the antioxidant activity of terpenes from 17 different sponges: *Psammocinia* sp. [16]; *Sarcotragus spinulosus* [15]; *Didiscus aceratus* [14]; *Dysidea* sp. [14]; *Haliclona* sp. [17]*; Hyrtios* sp. [22]; *Aka coralliphaga* [23]; *Sponginella* sp., [28]; *Dactylospongia* sp. [24]; *Hippospomgia* sp. [25]*; Dysidea fragilis* [19]; *Hyrtios erecta* [29]; *Dysidea avara* [26]; *Spongionella Gracillis* [18]; *Verongula rigida*, *Smenospongia cerebriformis* and *Smenospongia aurea* [27]. Regarding sponge orders the most classification studied was Dictyoceratida, with 12 articles reporting antioxidant activity [14–16, 18, 19, 22, 24–27, 29], followed by Hapclerida listed in 2 articles [17, 23] and Halichondrida, Dendroceratida and Verongiida described in 1 study each [15, 27, 28]. Regarding the classification of families: Thorectidae was the most studied (reported in 4 articles) [22, 24, 25, 27]; Dysideae was described in 3 articles [14, 19, 26]; 2 articles described Irciniidae [15, 16], and Dictyodendrillidae were studied in 2 articles [18, 28]. Others studies have investigated terpenes in the Desmoxcyidae, Chalinidae, Phloeoductyidae, Spongiidae and Aplysinidae families [15, 17, 23, 25, 27].

Terpenes are classified according to the number of isoprene units (Additional file 1: Fig. S2), about the class of terpenes, 7 studies used sesquiterpene compounds [14, 15, 19, 22, 23, 26, 27], 3 studies found sesterterpene [16, 25, 29], 2 studies reported the presence of diterpene [18, 28], 1 study obtained meroditerpene and another study found meroditerpenoid [17, 24]. Of the 37 terpenes included in this review, 3 were studied in more than one article: Puupehenone [14, 15, 24]; Ilimquinone [15, 27]; Heteronemim [25, 29] and Gacilin [18, 28]. The description of the site of sponge collection, the sponge genus and the classification of the terpenes identified together with their molecular structure are represented in the Additional file 1: Fig. S3 and Table S2. The extraction protocol was reported in 10 of 14 studies included. A total of 5 studies performed the extraction using methanol as a solvent [16, 17, 22–24], and 2 performed the extraction using methanol and dichloromethane solvents [28, 29], 1 study performed the extraction with ethanol [26], 1 study with ethyl acetate [25] and another used the chloroform for terpene extraction [15]. One study reported the use of a sonicator [22] and another study reported using the homogenizer for extraction [17, 25, 26]; 1 study did the elution with MeOH/H_2_O [16], 1 reported elution with methanol [23], 1 used *n*-hexane/EtOAC [29], 1 reported elution with chloroform [14]; 1 performed the elution with dichloromethane [22] and another realized the elution using hexane/acetone/MeOH/H_2_O [27] (Additional file 1: Table S3). Six studies did not describe the elution process [14, 18, 19, 24, 26, 28]

Table 1 describes the type assays performed to evaluate the antioxidative activity of extracted terpenes and the in vitro results. The measurement of intracellular ROS was the most frequently performed assessment among the studies, being performed in 6 of 17 included articles [18, 19, 25, 27–29] followed by scavenging activity of DPPH performed in 5 studies [14–17, 23]. Other analyzes were less frequently, such as: the antioxidant FARP test [24], Radical Scavenging activity; qualitative DPPH [22]; inhibition of linseed-oil oxidation; inhibition of rat brain homogenate lipid peroxidation [14]; Quantitative ORAC [22]; DNA replications [16]; estimation of GSH level [28], are-luciferase reporter gene assay; immunocytochemistry Nrf2; quantitative PCR [19]; NO determination [18] and Western Blotting [18, 19, 27, 28]. Seven studies performed a combination of tests to assess antioxidant activity [15, 16, 18, 19, 22, 27]. In the Western Blotting analysis, the most frequently studied protein expression was NrF2 [18, 19, 28], the other proteins studied were caspase 3 [28], HO-1 [19], INos, NOS, Cypa [18], SOD1 and SOD2 [27].

**Table 1:** In vitro analyses, overall results and outcomes of studies included

Authors	Antioxidant analysis (compounds tested)	Methodology	Results of antioxidant activity	Outcomes
Choi et al. 2004 [16]	Scavenging activity of DPPH (mixture of **7** and **8**) DNA replication (mixture **7** and **8**)	The activity of topoisomerase I was measured by the degree of relaxation of the super helical plasmid DNA	The mixture of compounds **7** and **8** showed moderate antioxidant activity The mixture of compounds **7** and **8** showed significant inhibition of DNA replication	+
Utkina et al. 2004 [15]	Scavenging activity DPPH (**11–17**) Inhibition of rat brain homogenate lipid peroxidation (**11–17**) Inhibition of linseed-oil oxidation (**1**, **14** and **15**)	The DPPH assay was performed in the final concentration at 50 µM	Compounds **11**, **14** and **16** had the antioxidant activity and the activity the compound **14** is comparable with that of α-tocopherol The compounds **13–15** retarded the auto-oxidation of linseed oil	+
Utikina et al. 2011 [14]	Scavenging activity DPPH (**13**;** 18** and **19**)	ND	Compound **19** exhibited the highest antioxidant activity in comparison with that compounds **13** and **18** and the standard antioxidant Trolox	+
Trianto et al. 2011 [17]	Scavenging activity of DPPH (**20**)	The DPPH was performed with Gallic acid and MeOH as positive and negative controls	Compound **20** exhibited antioxidant activity	+
Longeon et al. 2011 [22]	Qualitative DPPH screening (**21**) Quantitative ORAC (**21**)	The potential antioxidant activity of crude extracts and pure compounds were screening using the scavenging activity of the DPPH free radicals ORAC values are expressed as relative Trolox equivalent	Compound **21** showed antioxidant activity	+
Shubina et al. 2012 [23]	Scavenging activity of DPPH (**22–25**)	The assay was performed in different concentrations (10, 20, 50, 100 and 200 μM). Trolox was used for positive control	Compound **25** showed the higher antioxidant activity index that compounds **22**, **24** and **26**	+
Leróis et al. 2014 [28]	Measurement of intracellular ROS (**27–32**) Estimation of GSH level (**27–32**) Western blotting (**27–28**; **31–32**)	The ROS generation and estimation of GSH levels were performed with primary cortical neurons in compound concentrations at 0.1 and 1 μM The Nrf2 analysis by western blotting was performed with cortical neurons that were incubated with four compounds **27**, **28**, **31** and **32** for 6 h	Compounds **27**,** 29**,** 30** and **31** in concentration at 0.1 and 1 μM had decreased ROS. Compounds **28** and **32** decresead ROS only concentration at 0.1 μM Compounds **30** and **32** produced a significant increase in GSH if compared to neuron stress conditions Compounds **27**, **31** and **32** produced an increased expression of Nrf2 in the nucleus which means that the antioxidant pathway was activated	+
Hagiwara et al. 2015 [24]	FRAP antioxidant (**13** and **33**)	For this assay was then added 20 μL of sample in reaction FRAP, incubated for 8 min at room temperature and measured at 595 nm	Compounds **13** and **33** demonstrated antioxidant properties	+
Chen et al. 2018 [25]	Measurement of intracellular ROS (**34**)	The measurement ROS was performed in concentrations at 0.15 and 0.31 µg/mL	Compound **34** treatment decreased mitochondrial membrane potential; increased ROS level and induced Molt4 cell apoptosis	−
Liu 2018 [19]	Measurement of Intracellular ROS (**35–36**) Are-luciferase reporter gene assay (**35–36**) Western blotting (**35–36**) Immunocytochemistry Nrf2 (**35–36**) Quantitative PCR (**35–36**)	The skin keratinocyte cell line was treated with compounds at 10 μM for 6 h. next the cytosolic and nuclear protein extraction and ARE-luciferase reported gene assay were performed The measurement of intracellular ROS was performed with keratinocytes cell. The cells were incubated with the compounds at 2.5, 5 and 10 μM during 24 h	The compounds could exert cytoprotective effects by inhibiting the accumulation of ROS Treatment with compounds dose-dependently increased ARE luciferase activity Treatment of keratinocyte cells with compounds resulted in an increase of Nrf2 mRNA Immunocytochemistry analysis verified the accumulation of Nrf2 in the nucleus	+
Cheng et al. 2018 [29]	Measurement of intracellular ROS (**34**) Mitochondrial Membrane potential (**34**)	ROS measurement and mitochondrial membrane potential were performed with lung cancer cells by flow cytometry. The cells were incubated with compounds at 0.15 and 0.31 μg/mL during 24 h	Compound **34** potently induced excessive oxidative stress through the inhibition of antioxidants, and a mitochondrial membrane disruption	−
Nakarada et al. 2020 [26]	Radical Scavenging activity (**37**)	Scavenging activity was performed with O_2_, NO and ASC radicals	Compound **37** was a potent antioxidative activity towards OH radicals, followed by Asc, O_2_ and NO	+
Gegunde et al. 2019 [18]	Measurement of Intracellular ROS (**31**) NO Determination (**31**) Western Blotting (**31**)	The measurement ROS, NO determination and Western blotting were performed with microglia cells in concentrations of compounds at 0.01, 0.1 and 10 μg/mL	In stress conditions compound **31** mitigated the production of cytokines, ROS and NO that injured the neurons causing their death In stress conditions, the production of NO decreased. Pre-treatment with compound **31** over microglial cells resulted in a decrease of CypA expression	+
Bajpai et al. 2022 [27]	Measurement of intracellular ROS (**11**) Western blotting (**11**)	The measurement ROS and Western blotting were performed with fibroblast cells in concentrations of compounds at 1, 5 and 10 μg/mL	Compound **11** was natural cellular antioxidant that effectively scavenged the harmful ROS and showed a positive impact on antioxidant enzymes Fibroblastic cells treated with compound **11** had higher expression SOD1 and SOD2	+

*DNA* deoxyribonucleic acid, *DPPH* 2,2-diphenyl-1-picrylhydrazyl, *MeOH* methanol, *ORAC* oxygen radical absorbance capacity, *ROS* reactive oxygen species, *GSH* glutathione, *Nrf2* nuclear factor E2-related factor 2, *FARP* ferric reducing antioxidant power, *Molt4* leukemia cell line, *OH* hydroxyl, *Asc* ascorbyl radical, *O2* superoxide anion, *Cypa* cyclophilin A, *SOD2* superoxide dismutase2, *SOD1* superoxide dismutase 1

Eight studies described the concentration of terpenes evaluated in antioxidant assays [15, 18, 19, 23, 25, 27–29], on the other hand 6 studies had not reported the tested concentrations [14, 16, 17, 22, 24, 26]. The studies used different measurement units to refer to the concentrations, five studies reported the concentration used in µM and another 3 in µg/mL. In studies that reported the concentrations in µM, the lowest concentration used was 0.01 µM [29] and the highest concentration studied was 200 µM [23]. In the studies that reported the concentration in µg/mL, the lowest concentration used was 0.01 µg/mL [18] and the highest concentration studied was 10 µg/mL [27].

**Fig. 1:**
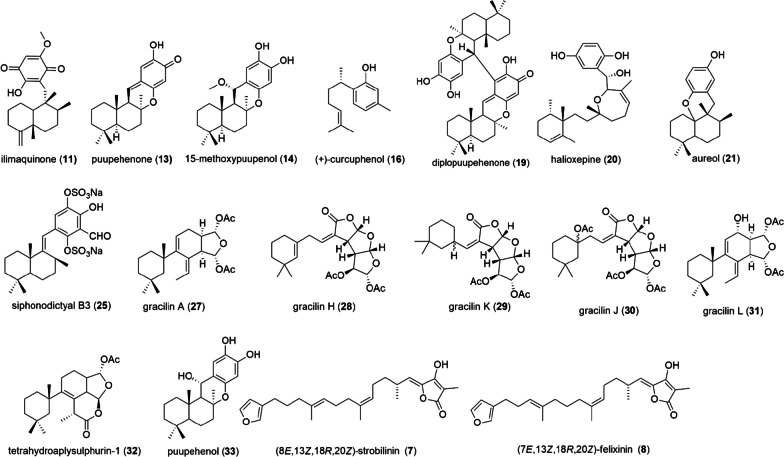
Molecular structures of terpenes with related antioxidant activity

Among the compounds studied (Additional file 1: Table S2) those that showed antioxidant activity were: compound **11** [15, 27]; compounds **14** and **15** [15], compound **19** [14]; compound **20** [17]; compound **21** [22]; compound **25** [23]; compounds **27**–**32** [28]; compounds **13** and **33** [24]; compound **37** [26] and a mixture of compounds **7** and **8** [16] (Fig. 1). On the other hand, compound **34** treatment decreased mitochondrial membrane potential and increased ROS level in a leukemia cell line Molt4 cell [25], moreover this potently induced excessive oxidative stress through the inhibition of antioxidants and mitochondrial membrane disruption in lung cancer cells [29].

The main antioxidant changes evidenced in the included studies were: (i) decrease in the concentration of ROS [18, 19, 27, 28]; (ii) decrease in the expression of proteins such as Cypa [18], (iii) increase in GSH levels [28]; (iv) increase in the expression of proteins Nrf2 [28] SOD1, SOD2 [27] and in ARE luciferase activity [30]. Based on the possible mechanism of molecules and their relationship with structure features, few information was provided in the selected articles, highlighting some terpenes such as compounds **11**, **13**, **14**, **16**, **27**–**31**.

**Fig. 2:**
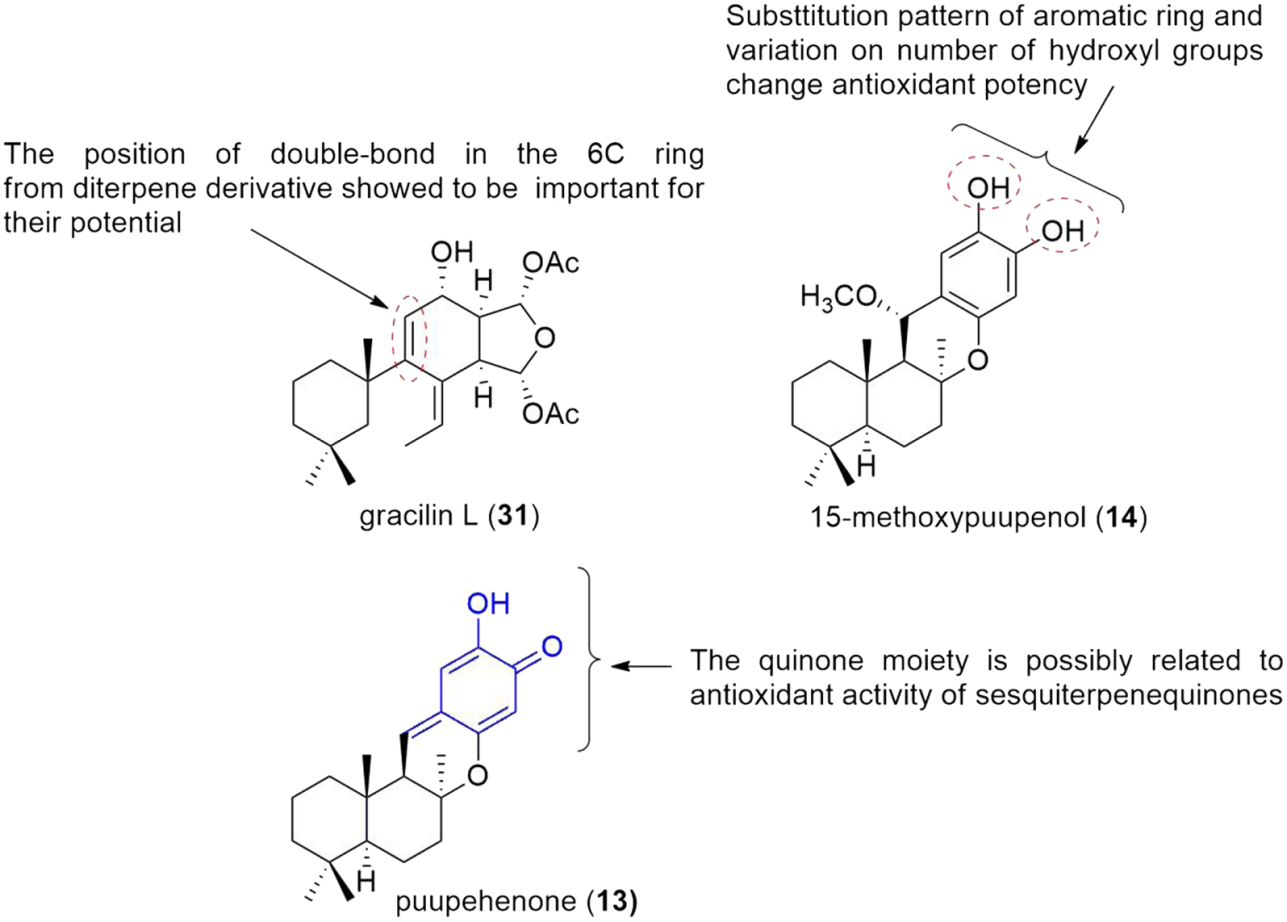
Potent terpenes-like antioxidants and structure features related to the antioxidant activity

Gracilins (**27**–**31**) are diterpene derivatives from *Spongionella* sp. with antioxidant activity by ROS reduction. Compounds **27**, **31** and **32** also showed to increase the expression of Nrf2 (Nuclear factor-erythroid 2-related factor 2)/ARE in the nucleus [28]. In addition, experiments revealed for compound **31** and synthetic derivatives a decrease of cyclophilin (Cypa) expression as well, associated to anti-inflammatory and neuroprotective effect [18]. Insights on structure and their potential as immunosuppressive, anti-inflammatory and antioxidant, was made evaluating gracilin L (**31**) and synthetic derivatives, pointing out functional groups which may be important for activities, likewise the double-bond in the 6C ring. Diterpene derivative with similar structure differing only in the double-bond position in the 6C ring decrease potency (Fig. 2) [18].

The study of sesquiterpenes, with quinone or phenol moiety, isolated from marine sponges and semisynthetic derivatives revealed compounds **11**, **13**, **14**, **16** with antioxidant activity and structure activity relationship (SAR) was described [15]. Compound **14** with phenol moiety showed expressive antioxidant activity (IC_50_ of 9.9 µM), probably due to two ortho-hydroxyls in the aromatic ring, since potential of phenolic compounds is linked to number and position of hydroxyl groups (Fig. 2) [15]. The puupehenone (**13**), sesquiterpenequinone (IC_50_ of 6.9 µM), was the most potent among compounds evaluated probably due to high antiradical activity [15]. Puupehenone showed antitumor and antibacterial activities as well [14, 15, 24].

It is well known that quinone core undergoes reversible reduction reaction to hydroquinone through Fe^2+^ ion (initiate radical-formation process) and sesquiterpenequinones antioxidant activity may be due to their ability to oxidize Fe^2+^ ions to Fe^3+^, suppressing radical formation [15]. Additionally, ilimaquinone (**11**) reduced expressively ROS and its antioxidant effect were further investigated for mechanism understanding. Compound **11** showed to influence positively on the expression of antioxidant proteins, superoxide dismutase 1 (SOD1) and superoxide dismutase 2 (SOD2) in fibroblast cells, suggesting the role which ilimaquinone prevents oxidative stress [27]. In addition, antioxidant and antibacterial potential of ilimaquinone could be linked to their structural configuration and the presence of its quinone moiety [27].

Additional assays were performed with terpenes and the results of the articles are described in this review (Additional file 1: Table S4). Nine studies performed additional analysis including assays to measure the cell viability [16–19, 22, 25, 27–29]. In addition, other analyses were performed in two studies: ATP concentration [27, 29]; apoptosis cells [25, 29]; and mitochondrial membrane potential [18, 25]. Other analyses were performed in only 1 study each: DNA fragmentation assay [25]; mitochondrial function [28]; determination of cytosolic calcium concentration [28]; measurement of caspase 3 [28]; PLA2 inhibition [22]; measurement of IL6 and TNF-a [18]; antimicrobial assay [24]; microbial susceptibility, bacterial killing kinetics, microbial cell morphology and cellular damage, K+ ion assay and genetic components [27].

Five studies performed viability assay only with cancer cells [16, 17, 22, 25]; 4 articles accomplished their research with non-cancerous cells [18, 19, 27, 28] and 1 study compared cancer cells with non-cancerous cells [29]. The cancer cells used were: lung [16, 29]; glioblastoma [16, 29]; ovary [16]; skin [16]; colon [16, 25]; urinary cancer cell [17]; pharyngeal [22]; breast [25]; prostate [25]; leukemia [25]; hepatoma [29]. The non-cancer cells used in the studies included were: Cortical Neurons [28]; keratinocytes [19]; microglia [18] and fibroblast [27, 29].

In the viability of cancer cells of the compounds included in this review it was found that: compounds **1**–**10** showed cytotoxicity against skin cancer cells [16]; compound **20** has cytotoxicity against urinary cancer cells [17]; compound **21** exhibited cytotoxicity on human pharyngeal carcinoma cells [22] and compound **34** showed cytotoxic effect in leukemia [25]. Cheng et al. [29] showed that compound **34** inhibited the cell viability of the cancer cells but there was no influence on non-tumor cells. In non-cancerous cells it was shown that: compounds **35** and **36** demonstrated a protective effect on keratinocyte cells [19]; compound **11** has also been shown to have a protective effect on fibroblast cells [27] while compound **31** did not show toxic effect on microglia cells [18] as well as compounds **27**–**32** as well they all are not cytotoxic in cortical neurons [28].

Regarding cellular mechanisms, it was identified that compound **34** decreased ATP production in lung cancer cells [29], while the compound **11** provoked massive release of extracellular ATP in both *S. aureus* and *E. coli* [27]; compound **31** inhibited the increase of mitochondrial membrane potential [18] and compound **29** did not show neuroprotection effects at mitochondrial function level [28] the compound **34** treatment decreased mitochondrial membrane potential and triggered DNA fragmentation [25].

On the mechanisms of cell death, it was possible to identify that the compounds **27** and **31** inhibited the enzymatic activity of caspase-3 [28]; while compound **34** induced the apoptosis in lymphoblastic cell and lung cancer cells [25]. In addition, downregulated Bcl-2 protein had an increase in the expression of Bax protein [29]

In turn, it was also possible to identify that compounds **13** and **33** were active against Gram-positive bacteria Staphylococcus aureus and Bacillus cereus [24] and that compound **11** showed antibacterial efficacy against *S. aureus* and *E. coli*. besides of causing evident cell damage due to bacterial cell wall rupture and altered cell morphology [27].

## Discussion

In recent years, there has been a growing interest in substances isolated from marine organisms. Attention has focused on bioactive compounds and secondary metabolites, because these substances have a variety of beneficial pharmacological effects. Concerning our goal, we performed a systematic review using the available data in the literature evaluating the antioxidant effects of terpenes from marine sponges.

According to the publications, different locations of marine sponge collection were used by the different researchers mainly in Asia, but also in Europe and North America. The terpenes were extracted from 18 different marine sponge species. It is interesting to mention that the *Dictyoceratida* was the order most evaluated. *Dictyoceratida* (Phylum Porifera, Class *Demospongiae*, Order *Dictyoceratida*) has been divided into five distinct families: *Dysideidae*, *Irciniidae*, *Spongiidae*, *Thorectidae*, and *Verticilliitidae*. Furthermore, Dictyoceratida order is the widely distributed geographically where they were reported in 31 countries. The major contributors were Korea, Japan, Australia, China, Papua New Guinea and Indonesia. Besides, this order is enormously rich in terms of chemical diversity of their metabolites. In addition, the order *Dictyoceratida* has contributed over 20% of new secondary metabolites which were previously derived from all sponges, making it the highest producer among all the sponge orders [31].

Concerning the terpene extraction methods, different solvents were applied, and the methanol extraction was the most used. High Performance Liquid Chromatography (HPLC) and vacuum flash chromatography were used for terpene isolation. These are common and basic methods of extraction and isolation of terpenes, and combinations of basic protocols can be used to optimize extraction and isolation of the desired terpenoid.

The methodological quality of the studies included in this review was accessed by the Grade Program, which offers a transparent and rigorous process for the development and presentation of summaries of evidence for systematic revisions. After this analysis, the studies included were characterized as moderate quality, which indicates that the evaluated works have strong scientific evidence [32]. However, some works did not fully answer the questions defined in this systematic review. The main questions not addressed by the studies were: (i) detailed statistical description; (ii) appropriate methodological information and (iii) inclusion of positive control in the experiments performed. Further studies should, therefore, have greater reliability and detail in the description of their methods and incorporate the use of positive control groups in their experimental designs.

In the studies evaluated, the main in vitro assays were cell viability, neuroprotection assessment, mitochondrial function, calcium concentration, cell apoptosis quantitative PCR and apoptosis. Furthermore, all analyses demonstrated antioxidant effects of the investigated terpenes and no cytotoxicity. Moreover, the antimicrobial activity for *Staphylococcus aureus* and *Bacillus cereus* and the antibacterial efficacy against *S. aureus* and *E. coli* were also reported.

Oxidative stress (OS) is a key pathological process in acute and chronic diseases [30]. Free radicals are highly active molecules produced during cellular respiration and normal metabolism, being of the products of these activities the production of reactive oxygen and nitrogen species (RONS) [33]. OS appears from the imbalance between antioxidant defenses and excessive RONS production. At low levels, RONS act for signaling molecules which regulate cell growth and proliferation [34]. On the other hand, a high concentration of RONS provokes damage to cells and tissues. Therefore, exogenous antioxidant substances may be used as pharmacological therapeutic agents [35].

Scavenging activity of DPPH, intracellular ROS, FRAP antioxidant, cytoprotective activity, flow cytometry analysis ROS and western blotting were positively applied in antioxidant analysis or the determination of the antioxidant capacity of the terpenes. Most methodologies may reveal pro-oxidant activities as well. As a mentioned, terpenes have a great chemical diversity and as a consequence a numerous number of possible mechanisms of action. Thus, there is not yet a universal method for antioxidant capacity screening.

It is worthwhile to emphasize that almost all the studies showed positive outcomes obtained from the investigated terpenes, except the studies performed by Chen et al. where the compound **34** demonstrated pro-oxidant effect, induced by excessive OS through the inhibition of antioxidants. This effect was observed, by Chen et al., through increased levels of ROS level which led to a cytotoxic effect for a leukemia cell line (Molt4 cell). A similar effect was reported in other study, in the lung cancer cells treated with compound **34** was observed an increase in cellular ROS, which in turn caused the loss of mitochondrial membrane potential [25, 29].

Interestingly, some terpenes compounds **11** and **13** have been identified in more than one species of sponges, thus demonstrating their great abundance. The compound **11** is a sesquiterpene quinone isolated from *Verongula rigida*, *Smenospongia cerebriformis*, and *Smenospongia aurea* sponges, demonstrating excellent antioxidant activity observed by in the reduction of intracellular ROS and DPPH assay [27, 36]. Additionally, compound **11** was also described in the literature as isolated from sponge *Hippiospongia metachromia*. This terpene exhibits anti-bacterial, antiviral, and anti-cancer activities [36–38]. On the other hand, compound **13**, identified in the sponges *Didiscus aceratus*, *Sarcotragus spinulosus*, *Dysidea* sp. and *Dactylospongia* sp. have not shown an important antioxidant activity. The literature describes a diverse effect of this sesquiterpene quinone, including antiangiogenic, antitumoral, antioxidant, antimicrobial, immunomodulatory and antiatherosclerotic effects [39].

The antioxidant activity of some terpenes was evaluated in experimental models of cancer and neurodegenerative diseases. Importantly, the terpenes identified by Leiróis et al. are interesting candidates for drug development of neurodegenerative diseases, such as Parkinson’s, Alzheimer’s, Friedreich ataxia or Amyotrophic lateral sclerosis [28] since these compounds **27**–**32** showed a neuroprotective effect, and neurodegenerative diseases are closely associated with OS.

Regarding chemical nature, several classes of terpenes were found, among them the most studied was the class of sesquiterpenes, with 20 identified compounds. Sesquiterpenes have low volatility and great potential for stereochemical diversity. Structure activity relationship of diterpene derivatives and sesquiterpenes allowed to point out some structure features which influenced on antioxidant potential of molecules, likewise the quinone and phenol moiety on sesquiterpenes. Moreover, these chemical entities can be a rich reservoir of candidate compounds for drug discovery [40]. Mostly of sesquiterpenes, diterpene derivatives and serterterpene described in this review with antioxidant activity demonstrated other biological potentials, such as antimicrobial [24, 27], immunosuppressive, anti-inflammatory [18], anti-cancer [17, 22, 25], anti-bacterial, antiviral [36–38] and neuroprotective activities [18, 28]. Antioxidants are well known to be related to anti-cancer [13] and neuroprotective effect [18, 28]. The neuroprotectors have the ability to up-regulate antioxidant enzymes reducing ROS [28]. Gracilins in face of inflammatory process were able to activate anti-oxidative mechanism, probably by CypA modulation [18]. In addition, a review published by Bartikova et al. showed that many of sesquiterpenes biological activities are based on antioxidant action, which is in agreement with the findings of this systematic review [40].

Finally, in view of the high failure rate in the translation of preclinical results to clinical trials in chronic diseases, it is important to evaluate the effects of the natural products such as terpenes that have shown promising pharmacological outcomes in vivo and in vitro studies.

## Conclusions

Marine sponge represents a large arsenal of bioactive products with antioxidant activity. Different classes of terpenes, such as sesterpene, sesquiterpenes, meroditerpene, diterpenes and meroterpenoid have been isolated and identified in the extracts of different sponges around the globe. The structural features of compounds can be an interesting core for the synthetic development of new candidates with antioxidant activity to treat a wide spectrum of diseases, including neurological disorders, bacterial, parasitic, viral, degenerative diseases, among others. More SAR study are needed for terpenes in order to straight compounds through specific targets and potentialize biological effects. Although some articles provide information of mechanism of action in vitro and in vivo, for a better understanding further investigation must be made concerning to correlations between antioxidant compounds and their interference in metabolic pathways inducing others pronounced pharmacological activities. The collected preclinical evidence in this systematic review may be helpful in planning future research as well providing information of terpenes as candidate compounds for drug design and other industrial applications.

## Methodology

### Review protocol

The review of articles was performed in April of 2022 in the databases Embase, PubMed and Scopus. The search was carried out according to the orientations of Preferred Reporting Items for Systematic Reviews and Meta-Analysis (PRISMA). The search of articles was performed using the descriptors shown in Table 2. Furthermore, to perform a broad search, synonyms and truncated terms of the descriptors were added in the search strategy. The search strategy was modified for use according to the guidelines of the searched database.

**Table 2:** Search strategy containing all terms and synonyms retrieved in the databases

Terms	
Component 1:	“porifera” OR “marine sponge*” OR “sponge*”
Component 2:	“terpen” OR “isoprenoid” OR “sesquiterpen*” OR “diterpen” OR “sesterpen*” OR “monoterpen*” OR “tetraterpen*” OR “hemiterpen*”
Component 3:	“antioxidant*” OR “antioxidantlevel*” OR “selenium” OR “carnitine*” OR “ascorbic acids” OR “zinc” OR “fatty acids” OR “flavonoid*” OR “L‐arginin*” OR “ubiquinol OR "l‐carnitin*” OR “beta‐caritine” OR “N‐acetylcysteine” OR “L‐acetyl‐carnitine” OR “acetyl L‐carnitine” OR “alpha tocopherol” OR “pentoxifylline” OR “inositol” OR “melatoni”

### Study selection

The title and abstract of the studies were analyzed separately by 2 reviewers (CCSM and BS) and the potential studies were identified from inclusion and exclusion criteria. Next, three reviewers (CCSM, BSS and ACMR) verified the eligibility of the selected studies. Disaccords were solved by discussion. The selected studies were further reviewed through a full-text screening, whereas studies that have not follow the eligibility criteria were excluded.

#### Inclusion criteria

1. Original articles
2. English Language
3. Articles published from 1987 to 2022
4. Analysis of in vitro antioxidant activity
5. Articles reporting the identification of terpene class from marine sponges

#### Exclusion criteria

1. Non-original articles
2. Languages different of English
3. Articles describing compounds from chemical classes other than terpenes.
4. Articles that evaluated only extracts
5. Studies that performed only in vivo methods
6. Terpenes of synthetic origin

### Data extraction

The antioxidant activity of the terpenes was analyzed by extracting data from different assays reported. The following variables were extracted: as author and year of publication; material collection location; sponge genus; extracted terpene; protocol for extraction and isolation of the terpene compound, chemical structure of compounds tested, in addition to the evaluations performed and results found.

### Types of described results

Evidence quality was determined using the GRADE approach, which analysis the following domains: trial design limitations due to risk of bias, inconsistency of results, indirectness, imprecision of results, and publication bias.

### Supplementary Information

**Additional file 1: Figure S1.** Flow diagram of literature search and selection criteria used in the present review. **Figure S2.** General classification of terpenes based on the number of isoprene units. **Figure S3.** Molecular structure of all terpenes compounds reported in the included papers from this systematic review. (A) sesterterpene, (B) sesquiterpenes, (C) other class. **Table S1.** Synthesis of scientific evidence: GRADE. **Table S2.** Description of the collection site, sponge genus and identified terpene compounds. **Table S3.** Description of the extraction and isolation protocol of the terpenes. **Table S4.** Description about additional analyses realized in the studies.
