# Supplementary material for: Terpenes extracted from marine sponges with antioxidant activity: a systematic review
Source: Nat Prod Bioprospect. 2023 Aug 9;13(1):23. doi: 10.1007/s13659-023-00387-y (PMC10409963; doi:10.1007/s13659-023-00387-y)
Supplement: Supplementary file 1 — Additional file 1: Figure S1. Flow diagram of literature search and selection criteria used in the present review. Figure S2. General classification of terpenes based on the number of isoprene units. Figure S3. Molecular structure of all terpenes compounds reported in the included papers from this systematic review. (A) sesterterpene, (B) sesquiterpenes, (C) other class. Table S1. Synthesis of scientific evidence: GRADE. Table S2. Description of the collection site, sponge genus and identified terpene compounds. Table S3. Description of the extraction and isolation protocol of the terpenes. Table S4. Description about additional analyses realized in the studies. [file 13659_2023_387_MOESM1_ESM.docx]

# Additional file 1


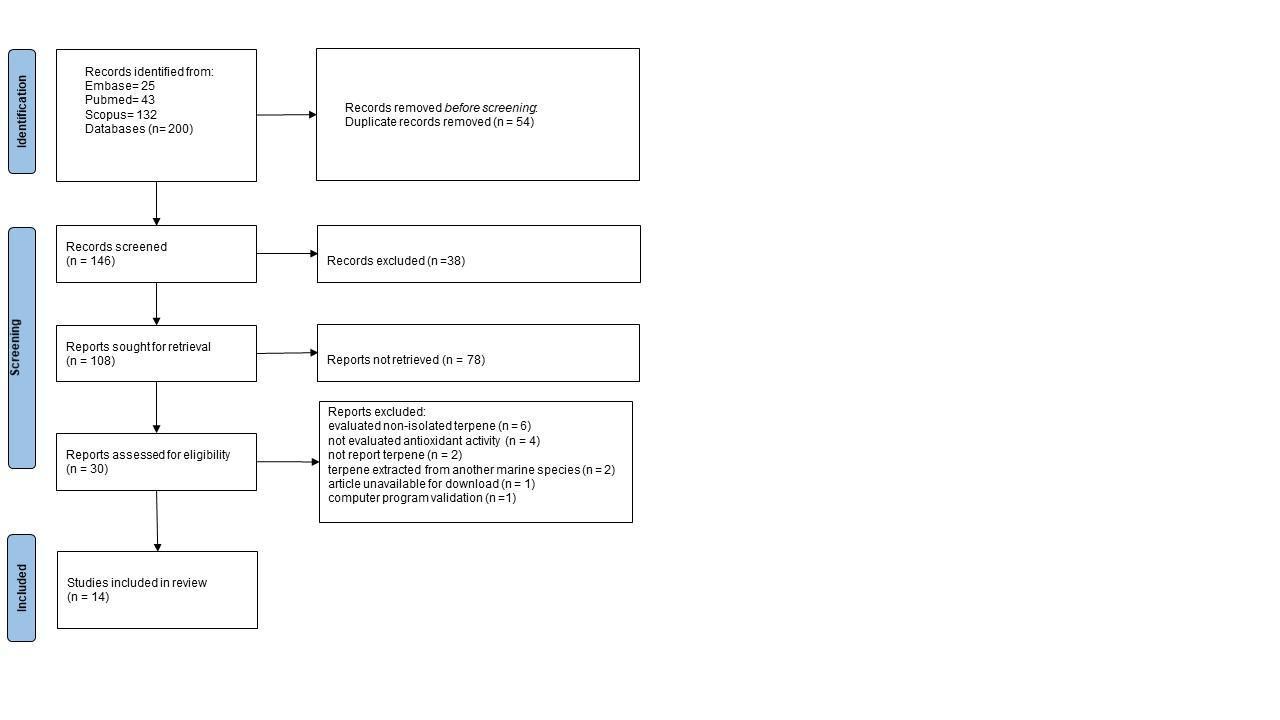


**Fig. S1** Flow diagram of literature search and selection criteria used in the present review


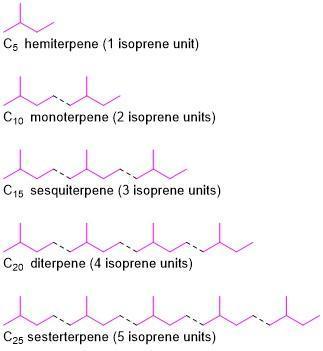


**Fig. S2** General classification of terpenes based on the number of isoprene units.

1. **Sesterterpene**

1. **Sesquiterpene**

1. **Other Classes**

**Fig. S3** Molecular structure of all terpenes compounds reported in the included papers from this systematic review. (A) sesterterpene, (B) sesquiterpenes, (C) other classes

**Table S1** Synthesis of scientific evidence: GRADE

| **Outcomes** | **Limitations** | **Inconsistency** | **Indirectness** | **Imprecision** | **Publication bias** | **Trials** | **Quality of the evidence (GRADE)** |
| --- | --- | --- | --- | --- | --- | --- | --- |
| **DPPH** |  |  |  |  |  |  |  |
|  | ✓ | ✓ | ^a^ | ✓ | ^c^ | Choi et al. 2004 |  |
|  | ✓ | ✓ | ✓ | ✓ | ✓ | Utkina et al. 2004 |  |
|  | ✓ | ✓ | ✓ | ^b^ | ^c^ | Utikina et al. 2011 |  |
|  | ✓ | ✓ | ✓ | ✓ | ✓ | Trianto et al. 2011 |  |
|  | ✓ | ✓ | ✓ | ✓ | ✓ | Longeon et al. 2011 |  |
|  | ✓ | ✓ | ✓ | ^b^ | ✓ | Shubina et al. 2012 |  |
|  |  |  |  |  |  |  | ⨁⨁⨁  MODERATE |
| **ROS** |  |  |  |  |  |  |  |
|  | ✓ | ✓ | ^a^ | ✓ | ^c^ | Leróis et al. 2014 |  |
|  | ✓ | ✓ | ✓ | ^b^ | ^c^ | Chen et al 2018 |  |
|  | ✓ | ✓ | ✓ | ✓ | ✓ | Liu 2018 |  |
|  | ✓ | ✓ | ^a^ | ✓ | ^c^ | Cheng et al. 2018 |  |
|  | ✓ | ✓ | ✓ | ✓ | ^c^ | Gegunde et al. 2019 |  |
|  | ✓ | ✓ | ^a^ | ✓ | ✓ | Bajpai et al. 2022 |  |
|  |  |  |  |  |  |  | ⨁⨁⨁  MODERATE |

✓: No Serious Limitations; ^a^: Absence of positive control; ^b^: poor statistical description; ^c^: Absence of methodological information;

**Table S2**  Description of the collection site, sponge genus and identified terpene compounds

| **Authors** | **Material Collection Location** | **Sponge Genus** | **Terpene extracted** |
| --- | --- | --- | --- |
| Choi *et al.* 2004 [16] | Ulleung Island, Korea | *Psammocinia sp.* (order *Dictyoceratida* Family Irciniidae) | **Sesterterpene** – (**1**) Psammocinin A1; (**2**) Psammocinin A2; (**3**) Psammocinin B; (**4**) Palinurin; (**5**) Isopalinurin; (**6**) (7E,12E,18R,20Z) variabilin; (**7**) (8*E*,13*Z*,18*R*,20*Z*) strobilinin; (**8**) (7*E*,13*Z*,18*R*,20*Z*) felixinin; (**9**) (8*Z*,13*Z*,18*R*,20*Z*) strobilinin and (**10**) (7*Z*,13*Z*,18*R*,20*Z*) felixinin |
| Utkina *et al.* 2004 [15] | Fiji sea and Scott reef in Australia.  (28°13′ S,175°23′4 E / 14°03′38 S, 121°46′67 E) | *Sarcotragus spinulosus* (order Dictyoceratida, Family Irciniidae) and- *Didiscus aceratus* (order Halichondrida, Family Desmoxyidae) | **Sesquiterpenes:** (**11**) ilimaquinone, (**12**) isospongiaquinone, (**13**) puupehenone, (**14**) 15-methoxypuupenol, (**15**) 2-methyl-2-pentaprenyl-6-hydroxychromene, (**16**) (+)-curcuphenol, (**17**) (+)-curcudiol |
| Utikina *et al.* 2011 [14] | ND | *Dysidea sp*. (order Dictyoceratida, family Dysideidae) | **Sesquiterpenes:** (**13**) Puupehenone, (**18**) Bispuupehenone, and (**19**) diplopuupehenone |
| Trianto *et al.* 2011 [17] | Baubau, Buton Island, South-East Sulawesi, Indonesia | *Haliclona sp*. (order Haplosclerida, Family Chalinidae,) | **Meroditerpene:** (**20**) Halioxepine |
| Longeon *et al.* 2011 [22] | Fiji Islands  (16°13.950’S, 179°01.900’E) | *Hyrtios sp*. (order Dictyoceratida, Family Thorectidae) | **Sesquiterpene:** (**21**) Aureole |
| Shubina *et al.* 2012 [23] | State Quintana Roo in southern Mexico | *Aka* coralliphaga (order Haplosclerida Family [Phloeodictyidae](https://www.marinespecies.org/aphia.php?p=taxdetails&id=131639)) | **Sesquiterpenes**: (**22**) siphonodictyal A sulfate, (**23**) akadisulfate A, (**24**) akadisulfate B, (**25**) Siphonodictyal B3 and (**26**) bis(sulfato)-cyclosiphonodictyol A |
| Leirós *et al.* 2014 [28] | ND | *Spongionella sp.* (order Dendroceratida Family Dictyodendrillidae) | **Diterpenes**: Gracilins (**27**) A, (**28**) H, (**29**) K, (**30**) J and (**31**) L) and (**32**) tetrahydroaplysulphurin-1. |
| Hagiwara *et al.* 2015 [24] | Hawaiian Island of Maui, (20°46.303′ N, 156°40.245′ W) | *Dactylospongia sp.* (order Dictyoceatidax Family Thorectidae) | **Meroterpenoid**: (**33**) Puupehenol and (**13**) puupehenone. |
| Chen *et al.*2018 [25] | Tai-tung coast, Taiwan | *Hippospomgia sp.* (order Dictyoceratida Family Spongiidae) | **Sesterterpene:** (**34**) heteronemim |
| Liu *et al.* 2018 [19] | ND | *Dysidea fragilis* (order Dictyoceratida  Family Dysideidae) | **Sesquiterpene aminoquinones:** (**35**) Dysidaminone H; (**36**) 3'-methylamino-avarone |
| Cheng *et al.* 2019 [29] | ND | *Hyrtios erecta* (order Dictyoceratida  Family Thorectidae) | **Sesterterpene:** (**34**) Heteronemin |
| Nakarada *et al.* 2020 [26] | Naples, Italy | *Dysidea avara* (order Dictyoceratida Family Dysideidae) | **Sesquiterpene:** (**37**) Avarol |
| Gegunde *et al.* 2020 [18] | ND | *Spongionella gracilis* (order Dendroceratida, Family Dictyodendrillida,) | **Diterpene:** (**31**) Gracilin L |
| Bajpai *et al.* 2022 [27] | Key Largo, FL, USA | *Verongula rigida* (order Verongiida Family Aplysinidae), *Smenospongia cerebriformis* (order Dictyoceratida Family Thorectidae), and *Smenospongia aurea* (order Dictyoceratida Family Thorectidae ) | **Sesquiterpene:** (**11**) Ilimaquinone |
| ND- Not described in the study | | | |

**Table S3** Description of the extraction and isolation protocol of the terpenes

| **Author** | **Method applied for extraction of compounds** | **Method applied for isolation of terpenes** |
| --- | --- | --- |
| Choi *et al.* 2004 [16] | The sponge was frozen, and the compounds were extracted MeOH. | The MeOH fraction was submitted to the reversed-phase flash column chromatography and eluted with a solvent system of MeOH/H_2_O. |
| Utkina *et al.* 2004 [15] | The sponge was lyophilized, and compounds were extracted with CHCl_3_. | The evaporated CHCl_3_ extracts were separated by vacuum flash-chromatography over a silica-gel column using hexane:ethyl acetate or CHCl_3_ and CH_3_OH. Fractions containing the phenolic compound were purified over a Sephadex LH-20 column using CHCl_3_. |
| Utkina *et al.* 2011 [14] | ND | The extract was chromatographed in an LH-20 Sephadex column in CHCl_3_**.** |
| Trianto *et al.* 2011 [17] | The compounds were extracted with MeOH. | The extract was triturated with CH_2_C_l2_ to provide a lipophilic fraction. After passing through column silica gel with EtOAc, the fraction was submitted to silica HPLC with a mixture of n-hexane and EtOAc. |
| Longeon *et al.* 2011 [22] | The sponge was lyophilized, and the terpenes were extracted with MeOH and sonication process for 15 min at room temperature | After solvent evaporation, 2 extracts were obtained (CH_2_C_l2_ and MeOH/H_2_O). An aliquot of the crude CH_2_Cl_2_ extract was chromatographed on a silica gel column, using a linear gradient of acetone in CH_2_Cl_2_ as eluent. |
| Shubina *et al.*. 2012 [23] | Extraction of terpenes was performed with MeOH. | After filtration and evaporation of the organic solvent in vacuo, the residue was submitted to column HPLC on Sephadex LH-20 using MeOH as eluent and YMC gel ODS-A using H_2_O with increasing amounts of MeOH to obtain fractions. The fractions eluted with MeOH were further purified by HPLC. |
| Leirós *et al.* 2014 [28] | The sponge was lyophilized, and the terpenes were extracted with MeOH and MeOH/CH_2_Cl_2_. | The extract was submitted to multiple steps of liquid/liquid fractionation, Sephadex LH-20 and RP-C18 chromatography to obtain the pure compounds. |
| Hagiwara *et al.* 2015  [24] | The sponge was lyophilized and compounds were extracted with CH_3_OH. | The extract was isolated by RP-HPLC |
| Chen *et al.* 2018 [25] | The sponge was lyophilized and the compounds were extracted with EtOAc. | The extract was separated by silica gel column in HPLC with n-hexane–EtOAc. |
| Liu *et al.* 2018 [19] | ND | ND |
| Cheng et al. 2019 [29] | The compounds were extracted with a mixture of organic solvent MeOH:CH_2_Cl_2._ | The extract was separated by silica gel column chromatography and then eluted with a mixture of n-hexane/EtOAc. |
| Nakarada *et al.* 2019[26] | The compounds were extracted with EtOH under homogenization for 3 hours. | The material was purified by HPLC. |
| Gegunde *et al.* 2019[18] | ND | ND |
| Bajpai *et al.* 2021 [27] | ND | The dried ethanol extract was submitted to silica gel vacuum liquid HPLC, followed by a sequence of gradient elution using hexane/ acetone/methanol/water. |

ND: data not descript in the study; MeOH: : Methanol; EtOAC: ethyl acetate; CHCl3: Chloroform; CH3OH: Methanol; CH2C12:dichloromethane; HPLC: High performance liquid chromatography; RP-HPLC: Reversed phase High Performance Liquid Chromatography; H_2_O: water; EtOH: etanol

**Table S4** Description about additional analyses realized in the studies

| **Authors** | **Additional analyses**  **(compounds tested)** | **Methodology** | **Results** | **Outcomes** |
| --- | --- | --- | --- | --- |
| Choi et al. 2004 [16] | Cell Viability (**1-6**; **7** and **8; 9** and **10**) | The compounds were evaluated for cytotoxicity in five human tumor cell line (A549; SK-OV-3; SK-MEL-2,; XF498 and HCT 15) | Compounds **1-6, 7** and **8; 9** and **10** showed slightly selective cytotoxicity against the SK-MEL-2, human skin cancer. | + |
| Trianto et al. 2011 [17] | Cell Viability (**20**) | The viability assay was performed against the urinary cancer cell. | Compound 20 showed moderate cytotoxicity against urinary cancer cell. | + |
| Longeon et al. 2011 [22] | Cell Viability (**21**)  PLA2 Inhibition (**21**) | The viability assay was performed with a human pharyngeal carcinoma cell line after 72 hrs. exposure in concentrations ranging from 1 μM to 100 μM.  For PLA_2_ inhibition assay was used 100 µg of the compounds. | Compound 21 showed cytotoxicity in human pharyngeal carcinoma cells and efficient PLA2 inhibitor | + |
| Leróis et al. 2014 [28] | Cell Viability (**27-32**)  Neuroprotection assays (**27-32**)  Mitochondrial function (**27-32**)  Determination of Cytosolic Calcium concentration (**27-28; 31-32**)  Measurement of Caspase 3 (**27-28; 31-32**) | The viability assay and mitochondrial function were performed with primary cortical neurons after 48 hrs. exposure compounds were tested at 0.01, 0.05, 0.1 and 1 μM.  The neuroprotection assay was performed after 12 hr of incubation of compounds 1 and 0.1 μM with cells.  The determination of the cytosolic calcium concentration was performed with neuroblastoma cell. A total of four compounds 27- 28 and 31 – 32 were tested.  The measurement of caspase-3 was performed with cortical neurons and the apoptosis was induced during 6 hrs. | No cytotoxicity was observed in cortical neurons.  Compound **28** and **32** protected the integrity of neurons membrane in oxidative stress conditions.  Only compound **30** haven’t present neuroprotection effects at mitochondrial function level.  Compounds **27** and **32** increased the membrane potential variation with respect to neurons in stress conditions.  Compounds **31** and **32** were able to inhibit FCCP effect by 50%, whereas compound **27** was the least effective with a minimal inhibition.  Compounds **27** and **31** inhibited caspase-3 enzymatic activity significantly. | + |
| Hagiwara et al. 2015. [24] | Antimicrobial Assay (13 and 33) | Staphylococcus aureus, Bacillus cereus, Escherichia coli, and Pseudomonas aeruginosa with the concentrations at 1.0 and 0.1 mg/mL. | Compounds **33** and **13** are relatively active towards Gram-positive bacteria *Staphylococcus aureus* and *Bacillus cereus*. | + |
| Chen et al 2018 [25] | Cell viability (**34**)  DNA Fragmentation Assay (**34**)  Mitochondrial membrane potential (**34**)  Cells apoptosis (**34**) | Viability assay was performed with cancer cell line such as colon, breast, prostate, and leukemia for after 24 and 48 h of incubation with compounds.  The DNA fragmentation Assay, mitochondrial membrane potential and cell apoptosis were performed with Leukemia cell. | After 48 h, leukemia cell lines were more sensitive to the cytotoxic effect of compound **34**.  Compound **34** triggered DNA fragmentation  Compound **34** treatment decreased mitochondrial membrane potential; and induced lymphoblast cell apoptosis. | - |
| Liu 2018 [19] | Cell viability (**35-36**) | The viability assay was performed with keratinocytes cell, after 24 hr of incubation with compounds at 2.5, 5 and 10 μM. | The compounds exhibit cytoprotective effects in keratinocyte cell. | + |
| Cheng et al. [29] | Cell Viability (**34**)  ATP Concentration (**34**)  Apoptosis Detection (**34**) | The viability assay was performed with human lung carcinoma cells, human hepatoma cells, two human glioblastoma cell lines, human primary gingival fibroblast and oral mucosal fibroblast after 24 hrs. of incubation with compounds at 0.01, 0.1, 1 and 10 μM  The apoptosis detection and ATP concentration were performed with lung cancer cell for 24 hrs after incubation with compounds. | Compound **34** inhibited the cell viability of the cancer cells, but there is no influence on nontumor cells.  Compound **34** leading to a decrease of ATP production in lung cancer cells.  Compound **34** leaded to changes in the structure and content of the nuclear DNA and induced apoptosis in lung cancer cell. Bcl-2 protein was downregulated and an increase in the Bax protein expression in treatments of groups with 1 and 10 μM | - |
| Gegunde et al. 2020 [18] | Cell Viability (**31**)  Mitochondrial Membrane Potential (**31**)  Measurement of IL6 and TNF-a (**31**) | The viability assay, Mitochondrial membrane potential assay and measurement of IL6 and TNF-a level were performed with microglia cell, after 24 hr of incubation with compounds at 0.01, 0.1, 1 and 10 μM. | Non-cytotoxic effects in microglia cell were observed in compounds up 1 μM.  Compound **31** inhibits the increase of mitochondrial membrane potential  The compounds suppress pro-inflammatory cytokines production in microglia. | + |
| Bajpai et al. 2022 [27] | Cell viability (**11**)  Microbial susceptibility and Bacterial killing kinetics (**11**)  Microbial cell morphology and cellular damage (**11**)  K^+^ ion assay (**11**)  Release of extracellular ATP (**11**)  Genetic components **(11**) | The viability assay was performed with *Escherichia coli*, *Staphylococcus aureus* and Fibroblast.  The Microbial susceptibility was tested with Bacteria cultures of *Escherichia coli* and *Staphylococcus aureus* in concentrations at 250, 125, 62.5, 31.25, 15.62 and 7.81 μg/mL. incubated for 24 hrs.  The bacterial killing kinetics was estimate in the different time intervals 0, 40, 80, 120, 160 and 200min. | Antibacterial efficacy against *S. aureus* and *E. coli* with minimum inhibitory concentration values of 125 and 250 μg/mL.  Evident cellular damaging effect by the ruptured bacterial cell wall and altered cellular morphology    Significant release of K^+^ ions from *S. aureus* and *E. coli* in a time-dependent manner.  Massive release of extracellular ATP in both *S. aureus* and *E. coli*  Compound **11** provoked the release of genetic material from bacterial cells. | + |

K+: Potassium; ATP; adenosine 5′-triphophate; IL6: interleukin 6; SK-OV-3; human ovarian cancer; SK-MEL-2, human skin cancer; XF498, human CNS cancer; HCT 15, human colon cancer; A549: lung carcinoma cell; DNA: deoxyribonucleic acid; BAX- Bcl-2 associated protein X; Bcl2- B cell lymphoma protein 2; FCCP: phosphorylation uncoupler Carbonyl cyanide 4-(trifluoromethoxy)phenylhydrazone;
